# Phagocytic Mesothelial Cells in Pericardial Effusion Following Allogeneic Peripheral Blood Stem Cell Transplantation for Chronic Active Epstein–Barr Virus Infection: A Distinctive Cytomorphological and Immunocytochemical Case Study

**DOI:** 10.1002/dc.70094

**Published:** 2026-02-06

**Authors:** Hidetoshi Satomi, Ayumi Ryu, Sei Murayama, Hiroya Kojima, Satoshi Tanada, Keiichiro Honma

**Affiliations:** ^1^ Department of Diagnostic Pathology and Cytology Osaka International Cancer Institute Osaka‐City Osaka Japan; ^2^ Department of Clinical Laboratory Osaka International Cancer Institute Osaka‐City Osaka Japan

**Keywords:** cytology, immunocytochemistry, mesothelial cells, pericardial effusion, phagocytosis

## Abstract

Phagocytic activity of mesothelial cells is a rare phenomenon requiring careful differentiation from histiocytic hemophagocytosis to ensure appropriate patient management, as these phenomena could exhibit distinct clinical implications. In this study, we report a case of phagocytic mesothelial cells found in pericardial effusion that developed 18 months after allogeneic peripheral blood stem cell transplantation for chronic active Epstein–Barr virus (EBV) infection. The patient presented with fever and dyspnea, alongside mild chronic graft‐versus‐host disease, affecting the shoulders and skin. Cytological examination revealed mesothelial cells containing intracytoplasmic lymphocyte‐like structures and hemosiderin granules, confirmed by Berlin blue staining. Immunocytochemical analysis indicated pan‐cytokeratin (AE1/AE3), calretinin, and desmin positivity as well as CD163 negativity, confirming their mesothelial origin. The ferritin level of the patient remained at 370.0 ng/mL. EBV DNA was undetectable in peripheral blood. The clinical course was favorable, without systemic complications or recurrent effusion. This case highlights the importance of accurate cytomorphological and immunocytochemical characterization of phagocytic mesothelial cells in post‐transplantation settings.

## 1 Introduction

Mesothelial cells perform various biological functions, including inflammatory mediator production, antigen presentation, and tissue repair [1]. However, the phagocytic activity of mesothelial cells, particularly toward blood cells, remains poorly documented. The presence of intracellular hemosiderin‐like granules in phagocytic mesothelial cells suggests a potential physiological role in erythrophagocytosis [2]. Although histiocyte‐related hemophagocytosis is associated with poor prognosis under various pathological conditions, the clinical significance of mesothelial cell phagocytosis remains poorly understood [3, 4]. Differentiating between histiocytic hemophagocytosis and mesothelial cell phagocytosis is crucial for accurate diagnosis and appropriate patient management, as these processes could potentially display distinct clinical implications.

Herein, we present a case of pericardial effusion that developed upon allogeneic peripheral blood stem cell transplantation (allo‐PBSCT) for chronic active Epstein–Barr virus (CAEBV) infection, wherein the phagocytic activity of mesothelial cells was identified and immunocytochemically confirmed.

## 2 Case Report

A 3X‐year‐old man with adult‐onset CAEBV underwent allo‐PBSCT after receiving SMILE chemotherapy (dexamethasone, methotrexate, ifosfamide, l‐asparaginase, and etoposide). The patient developed graft‐versus‐host disease (GVHD) affecting the shoulder and skin, controlled with oral medication. Eighteen months post‐transplantation, he presented with fever (38.0°C) and dyspnea. Imaging revealed pericardial effusion, prompting pericardiocentesis.

**FIGURE 1:**
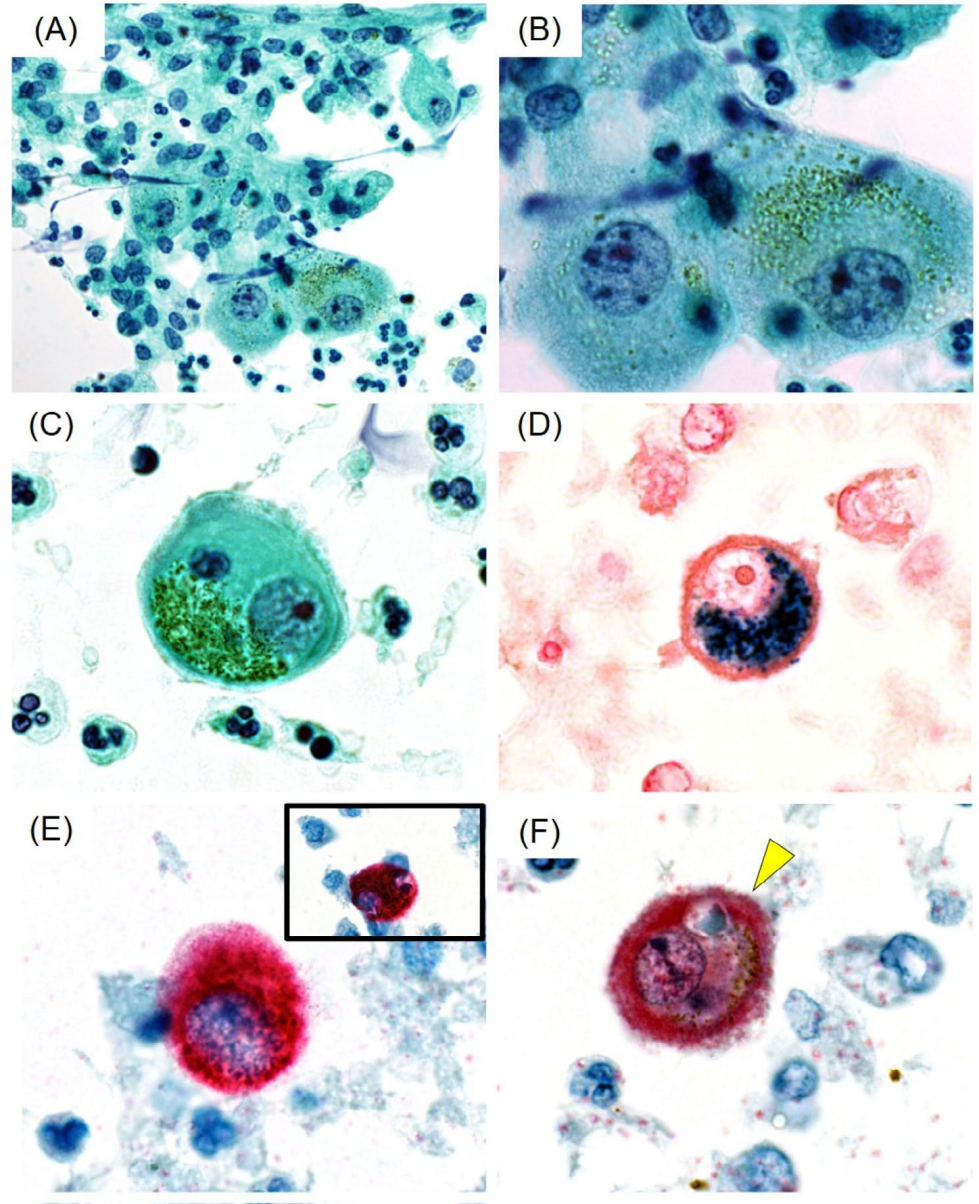
Cytomorphological spectrum of mesothelial cells in pericardial effusion. (A) Low‐power view presenting scattered distinct large cells with greenish‐brown granules among histiocytes and leukocytes. Erythrocytes are absent due to preanalytical hemolysis (Papanicolaou stain, ×400). (B) High‐power view demonstrating cells with indistinct cytoplasmic outlines, fine greenish‐brown granules, smooth nuclear membranes, abundant cytoplasm, and multiple distinct round nuclei (Papanicolaou stain, ×1000). (C) A mesothelial cell displaying classical cytomorphological features, including abundant cytoplasm, uniformly and intensely stained with the light green dye in Papanicolaou stain, centrally or paracentrally located nuclei with smooth membranes. Microvilli on the cell surface and focal granules could be noted in one cell (Papanicolaou stain, C: ×1000). (D) Berlin blue staining, highlighting hemosiderin as distinct blue granules (Berlin blue stain, ×1000). (E, F) Immunocytochemistry (ICC) performed on liquid‐based cytology specimens. The cells exhibited positive immunoreactivity for AE1/AE3 (E) and calretinin (F). The inset in (E) shows an intracytoplasmic lymphocyte‐like structure (E: AE1/AE3, ICC with alkaline phosphatase detection, ×1000; F: Calretinin, alkaline phosphatase detection, ×1000). These morphological and immunocytochemical findings confirm the presence of hemosiderin granule‐containing reactive mesothelial cells.

Cytological examination of the pericardial fluid revealed marked neutrophilic infiltration containing scattered large, round cells with an abundant cytoplasm (Figure 1A). The cytoplasm contained distinctive greenish‐brown granules, several cells exhibiting intracytoplasmic lymphocyte‐like structures. These cells displayed indistinct borders with microvilli‐like projections and nuclei lacking chromatin abnormalities (Figure 1B,C). Iron staining confirmed hemosiderin presence in the cells (Figure 1D). Immunocytochemically, these cells tested positive for pan‐cytokeratin (AE1/AE3), calretinin, and desmin (data not shown) and negative for CD163 (data not shown) (Figure 1E,F).

At presentation, Epstein–Barr virus (EBV) DNA and other active viral infections were undetectable in the peripheral blood. The peak ferritin level was 370.0 ng/mL (reference range: 21.0–282.0 ng/mL). The clinical diagnosis was non‐specific pericarditis. The patient showed no clinical deterioration, hematological malignancy recurrence, or recurrent pericardial effusion.

## 3 Discussion

Given the rarity of mesothelial cell phagocytic activity, particularly toward blood cells, careful cytological and immunocytochemical evaluation is required [5, 6]. In this study, we described a unique case demonstrating phagocytic mesothelial cells with distinctive cytomorphological and immunocytochemical features in pericardial effusion post‐allo‐PBSCT for CAEBV. This case expands our understanding of mesothelial cell behavior in immunocompromised patients and establishes diagnostic criteria for this rare but clinically significant phenomenon.

In this case, mesothelial cells exhibited notable features, including lymphocyte‐like structures and hemosiderin granules within their cytoplasm. The immunocytochemical profile (i.e., pan‐cytokeratin [AE1/AE3]‐, calretinin‐, and desmin‐positivity with CD163‐negativity) is crucial for confirming the mesothelial origin and differentiating these cells from histiocytes [7, 8]. This distinction is essential, as histiocytic hemophagocytosis typically indicates a severe systemic condition requiring urgent intervention, whereas mesothelial cell phagocytosis has not been associated with fatal outcomes [2].

Both the timing of this phenomenon, that is, 18 months post‐transplantation, and the clinical context are noteworthy. The ferritin level (370.0 ng/mL) of the patient was not markedly elevated, and EBV DNA was undetectable in the peripheral blood, suggesting no association with systemic hemophagocytic lymphohistiocytosis or EBV reactivation [9]. Instead, phagocytic mesothelial cells likely presented a localized response to the post‐transplantation immune environment or non‐specific inflammation [10].

Pericarditis and pericardial effusion are recognized cGVHD‐related complications following allogeneic hematopoietic stem cell transplantation, with reported incidence rates between 3.2% and 5% [11, 12]. These complications typically manifest within months to years post‐transplantation and frequently coincide with severe cGVHD involving multiple organs [9, 11]. In this case, while the patient developed mild GVHD manifestations in the shoulder within 18 months post‐transplant, the absence of severe systemic deterioration and the manageable nature of the lesions complicated the establishment of a definitive causal relationship among bone marrow transplantation, GVHD, and pericardial effusion. The observed pericardial fluid changes were likely attributable to non‐specific inflammatory processes.

Hemosiderin detection in the mesothelial cells, confirmed by iron staining, indicated local erythrophagocytosis [2]. These observations contribute to our understanding of the physiological role of mesothelial cells in tissue homeostasis and immune responses. While mesothelial cells could reportedly perform non‐specific phagocytosis, their specific function in post‐transplantation settings remains inadequately documented.

From a diagnostic perspective, distinguishing phagocytic mesothelial cells from malignant cells or histiocytes is crucial. Differential diagnoses should include reactive mesothelial cells, histiocytes with hemophagocytosis, and malignant cells. The absence of nuclear atypia, normal nuclear‐to‐cytoplasmic ratio preservation, and a characteristic immunocytochemical profile are pivotal for accurate identification [11, 13]. In this case, immunocytochemical findings were crucial for accurate categorization. Accurate identification of this phenomenon could prevent unnecessary invasive procedures and provide reassurance regarding its benign nature.

Table 1 summarizes the differential diagnosis and immunocytochemical characteristics of cells encountered in pericardial effusion specimens. This comprehensive comparison emphasizes the critical role of combined morphological and immunocytochemical assessment in accurately identifying phagocytic mesothelial cells and excluding other significant pathological entities.

**TABLE 1:** Differential diagnosis and immunocytochemical profile of cells in pericardial effusion.

	AE1/AE3	Calretinin	CK5/6	CD163	CEA	Additional markers
Mesothelial cells	+	+	+	−	−	Desmin+, CD68−
Malignant mesothelioma	+	+	+	−	−	Desmin−, WT1+, BAP1 loss
Histiocytes/macrophages	−	−	−	+	−	CD68+
Adenocarcinoma	+/−	−	−	−	+	TTF1, CK7/CK20, organ‐specific
Lymphoma cells	−	−	−	−	−	CD45+, CD20/CD3+

*Note:* Immunocytochemical results: + positive, − negative, +/− variable. The present case demonstrated phagocytic mesothelial cells displaying classic cytomorphological features, including abundant cytoplasm uniformly and intensely stained light green by Papanicolaou stain, centrally or paracentrally located nuclei with smooth membranes, and narrow intercellular spaces. Indistinct cytoplasmic outlines suggest well‐developed microvilli on the cell surface. Notable features include distinctive hemosiderin granules and a favorable clinical outcome.

Abbreviations: AE1/AE3, pan‐cytokeratin; BAP1, BRCA1‐associated protein 1; CEA, carcinoembryonic antigen; CK5/6, cytokeratin 5/6; CK7/CK20, cytokeratin 7/20; TTF1, thyroid transcription factor 1; WT1, Wilms tumor 1.

This study retains certain limitations. The spontaneous remission of pericardial effusion precluded sequential cytomorphological analysis, and the absence of standardized criteria hindered the quantitative assessment of mesothelial phagocytic activity. Similar cases should be investigated to elucidate potential relationships between mesothelial cell phagocytosis and the underlying clinical conditions. Detailed cytomorphological examination of pericardial effusion specimens emphasizing phagocytic phenomena might provide valuable insights for prognostication and therapeutic decision‐making in post‐transplantation care. The hereby‐established diagnostic framework provides a reproducible approach for clinical practice. Future studies should focus on defining prevalence across different transplantation settings and establishing standardized diagnostic criteria to inform evidence‐based management.

In conclusion, the favorable clinical course of the patient, without systemic complications or recurrent pericardial effusion, suggests that mesothelial cell phagocytosis has clinical implications different from histiocytic hemophagocytosis. Although a single case cannot ensure a definitive prognostic significance, the hereby‐observed benign outcome highlights the need for further investigation into the biological and clinical characteristics distinguishing mesothelial cell phagocytosis from histiocytic hemophagocytosis. Such distinctions might significantly influence patient management in post‐transplantation settings. Future studies should focus on defining prevalence across different transplantation settings and establishing standardized diagnostic criteria to inform evidence‐based management strategies.

## Author Contributions

Hidetoshi Satomi was responsible for interpreting patient findings and manuscript preparation. Ayumi Ryu critically revised and approved the manuscript. Sei Murayama, Hiroya Kojima, and Satoshi Tanada approved the manuscript. Keiichiro Honma approved the final manuscript. All the authors have read and approved the final manuscript.

## Funding

The authors have nothing to report.

## Ethics Statement

This case report was approved by the Institutional Review Board at Osaka International Cancer Institute (no. 24132).

## Consent

Informed consent was obtained from the patient for publication of this case report and the accompanying images.

## Conflicts of Interest

The authors declare no conflicts of interest.

## Data Availability

The availability of the data used in this case study is subject to confirmation by the journal or the authors. For more information on data availability and access procedures, please contact the journal or corresponding author.
